# Primary glioblastoma with spontaneous transdural invasion and contiguous calvarial/mastoid osteolysis: a case report and literature review

**DOI:** 10.3389/fsurg.2026.1890926

**Published:** 2026-08-07

**Authors:** Zhijie Yang, Ling Ai, Jun Shen, Aibin Zhou, Shaolin Zhang

**Affiliations:** 1 Department of Neurosurgery, The First Affiliated Hospital of Wannan Medical University (Yijishan Hospital of Wannan Medical University), Wuhu City, Anhui, China; 2 Department of Neurology, The First Affiliated Hospital of Wannan Medical University (Yijishan Hospital of Wannan Medical University), Wuhu City, Anhui, China; 3 Department of Radiotherapy, The First Affiliated Hospital of Wannan Medical University (Yijishan Hospital of Wannan Medical University), Wuhu City, Anhui, China

**Keywords:** calvarial destruction, case report, glioblastoma, IDH-wildtype, mastoid osteolysis, skull-invasion, transdural invasion

## Abstract

**Background:**

Glioblastoma (GBM) is the most common and most aggressive primary malignant tumor of the central nervous system in adults. Although GBM is characterized by highly infiltrative growth within the brain parenchyma, spontaneous transdural extension with direct invasion of the calvarium and skull base at initial presentation is exceptionally rare. Such cases may radiologically mimic metastatic skull tumors or other extra-axial lesions, creating diagnostic and therapeutic challenges.

**Case presentation:**

We report the case of a 63-year-old man who presented with dizziness, slowed responses, and progressive gait impairment. Magnetic resonance imaging revealed a heterogeneously enhancing right temporal lobe mass with marked peritumoral edema, while computed tomography demonstrated contiguous osteolytic destruction of the right temporal calvarium and mastoid region. Magnetic resonance spectroscopy showed a markedly elevated choline peak, reduced N-acetylaspartate, and increased lipid/lactate peak, supporting a high-grade infiltrative glioma. The patient underwent gross total resection of the intracranial tumor together with resection of the involved skull lesion and cranioplasty. Histopathology confirmed glioblastoma, CNS WHO grade 4. Molecular testing demonstrated an IDH-wildtype profile with TERT promoter mutation, CDKN2A/CDKN2B homozygous deletion, PTEN loss, chromosome 10 loss, MGMT promoter unmethylation, and absence of 1p/19q codeletion. Postoperatively, the patient received radiotherapy to the tumor bed (60 Gy/30 fractions) and surrounding low-risk field (54 Gy/30 fractions) with concurrent temozolomide, followed by adjuvant temozolomide. Early postoperative MRI indicated gross total resection. At 3-month follow-up, the patient remained alive without radiographic progression, with relief of headache and no new neurological deficits.

**Conclusion:**

This case highlights an exceptionally rare presentation of primary IDH-wildtype glioblastoma with spontaneous transdural extension and contiguous calvarial/mastoid osteolysis before any cranial intervention. In addition to expanding the clinicoradiological spectrum of GBM, this case underscores the diagnostic value of integrating MRI, MRS, histopathology, and molecular profiling in skull-invasive lesions. Our literature review suggests that superficially located tumors with prolonged dural contact may facilitate transdural spread, whereas molecular alterations associated with aggressive mesenchymal-like behavior and extracellular matrix remodeling may contribute to osseous invasion. This report adds a contemporary molecularly characterized example to the limited literature on skull-invasive GBM.

## Introduction

Glioblastoma (GBM) is the most common malignant primary brain tumor in adults and remains associated with dismal prognosis despite multimodal treatment (1, 2). Its biological hallmark is diffuse infiltration of the surrounding brain parenchyma; however, spread beyond the dura and direct invasion of the skull are distinctly uncommon. In contrast to extracranial metastasis through hematogenous or lymphatic routes, spontaneous contiguous osseous invasion by untreated primary GBM is exceedingly rare (3–6). Because calvarial or skull-base destruction more commonly suggests metastatic disease, sarcoma, or invasive extra-axial tumors, such presentations may lead to diagnostic uncertainty and delayed recognition of the underlying intra-axial glioma.

Historically, only isolated reports have described glioblastoma with transdural extension and direct skull destruction (7–12). Many of these reports predate contemporary molecular classification and therefore provide limited insight into the biological basis of this invasive phenotype. With the advent of the 2021 World Health Organization (WHO) classification and integrated molecular diagnostics, it has become increasingly important to determine whether skull-invasive GBM merely represents an anatomic variant of conventional IDH-wildtype GBM or whether it may reflect a distinct biological propensity for extra-axial extension (14–17).

Here, we report an unusual case of primary right temporal glioblastoma presenting with spontaneous transdural invasion, contiguous temporal calvarial destruction, and mastoid osteolysis before any surgery, biopsy, or radiotherapy. We further integrate multimodal imaging, histopathology, and molecular profiling, and review previously reported cases of skull-invasive glioblastoma to better contextualize this rare presentation.

## Case presentation

A 63-year-old man presented with dizziness, slowed responses, and impaired mobility. His symptoms had progressively worsened over a short period, and family members noted reduced interaction and deterioration in gait. He had no history of cranial surgery, cranial trauma, or radiotherapy. Neurological examination on admission showed slowed responsiveness and impaired ambulation, without clear focal cranial nerve deficits. No scalp ulceration or overt extracranial mass was identified on initial physical examination.

**Figure 1:**
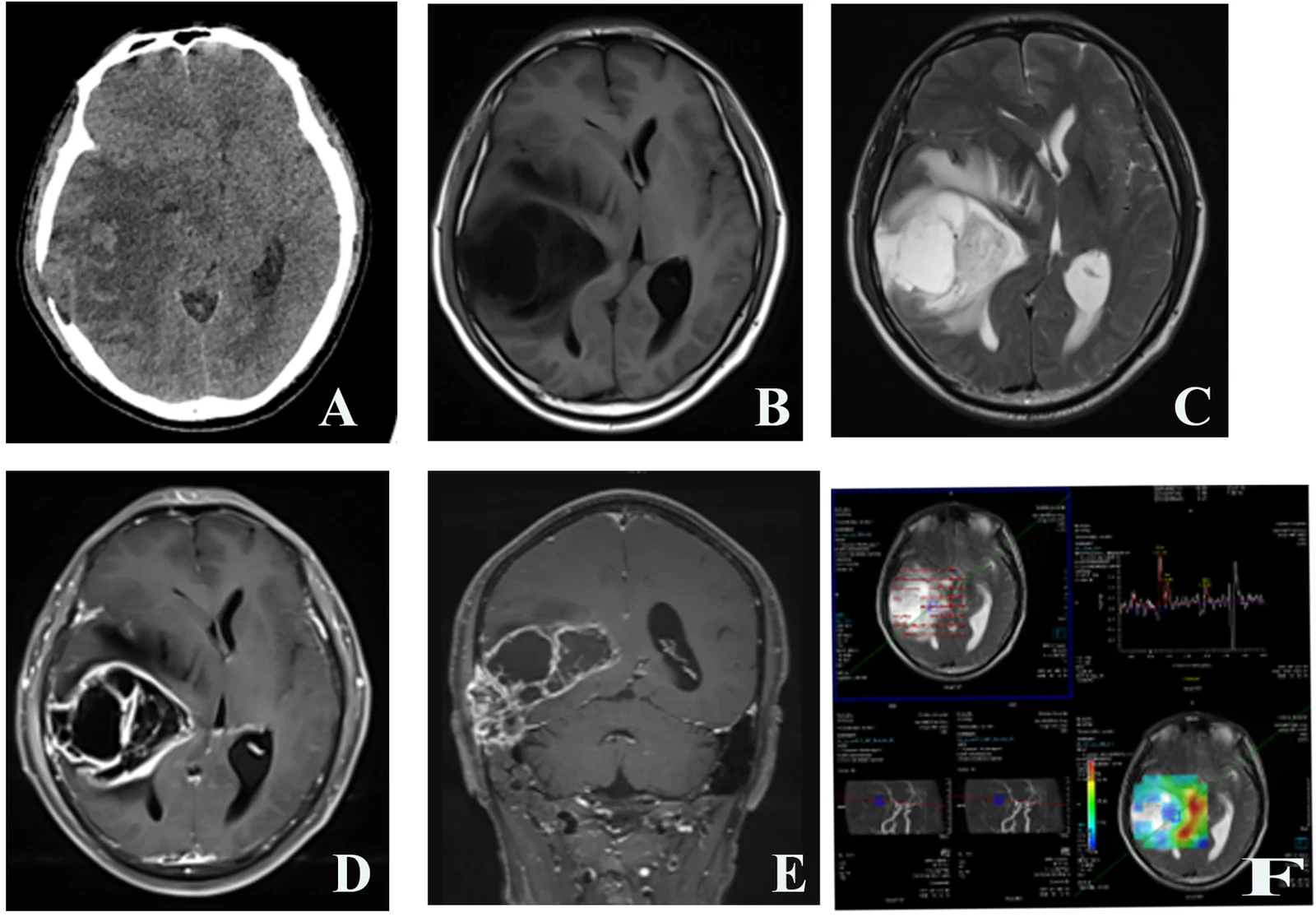
Preoperative CT, MRI and MRS of the patient. **(A)** Head CT demonstrated a mass lesion in the right temporal region, with adjacent osseous destruction and leftward midline shift. **(B)** Axial T1-weighted imaging (T1WI) showed the tumor as hypointense. **(C)** Axial T2-weighted imaging (T2WI) showed the tumor as hyperintense. **(D)** Axial contrast-enhanced T1WI revealed marked heterogeneous, ring-like enhancement of the tumor. **(E)** Coronal contrast-enhanced T1WI. **(F)** MRS showed a decreased NAA peak and an elevated Cho peak; the NAA/Cr ratio was 0.58, the Cho/Cr ratio was 2.46, the Cho/NAA ratio was approximately 4.21, and the lipid-lactate (LL) peak was elevated.

Brain magnetic resonance imaging (MRI) revealed a large irregular mass in the right temporal lobe with heterogeneous ring-like enhancement, extensive surrounding vasogenic edema, and mass effect. The lesion abutted the temporal dura and adjacent skull. Computed tomography (CT) demonstrated osteolytic destruction of the right temporal calvarium with involvement of the adjacent mastoid region, raising concern for direct skull invasion. The imaging appearance created a differential diagnosis that included high-grade glioma with extra-axial extension, metastatic skull lesion with intracranial invasion, and less likely primary skull malignancy (Figures 1A–E).

Magnetic resonance spectroscopy (MRS) further supported a high-grade infiltrative glial neoplasm, showing markedly elevated choline and reduced N-acetylaspartate, with Cho/NAA 4.21, Cho/Cr 2.46, and NAA/Cr 0.58, together with an elevated lipid/lactate peak (Figure 1F). This metabolic pattern was consistent with high cellular turnover, membrane synthesis, neuronal loss, and necrosis, all of which favored high-grade glioma over a benign or low-grade process (13).

**Figure 2:**
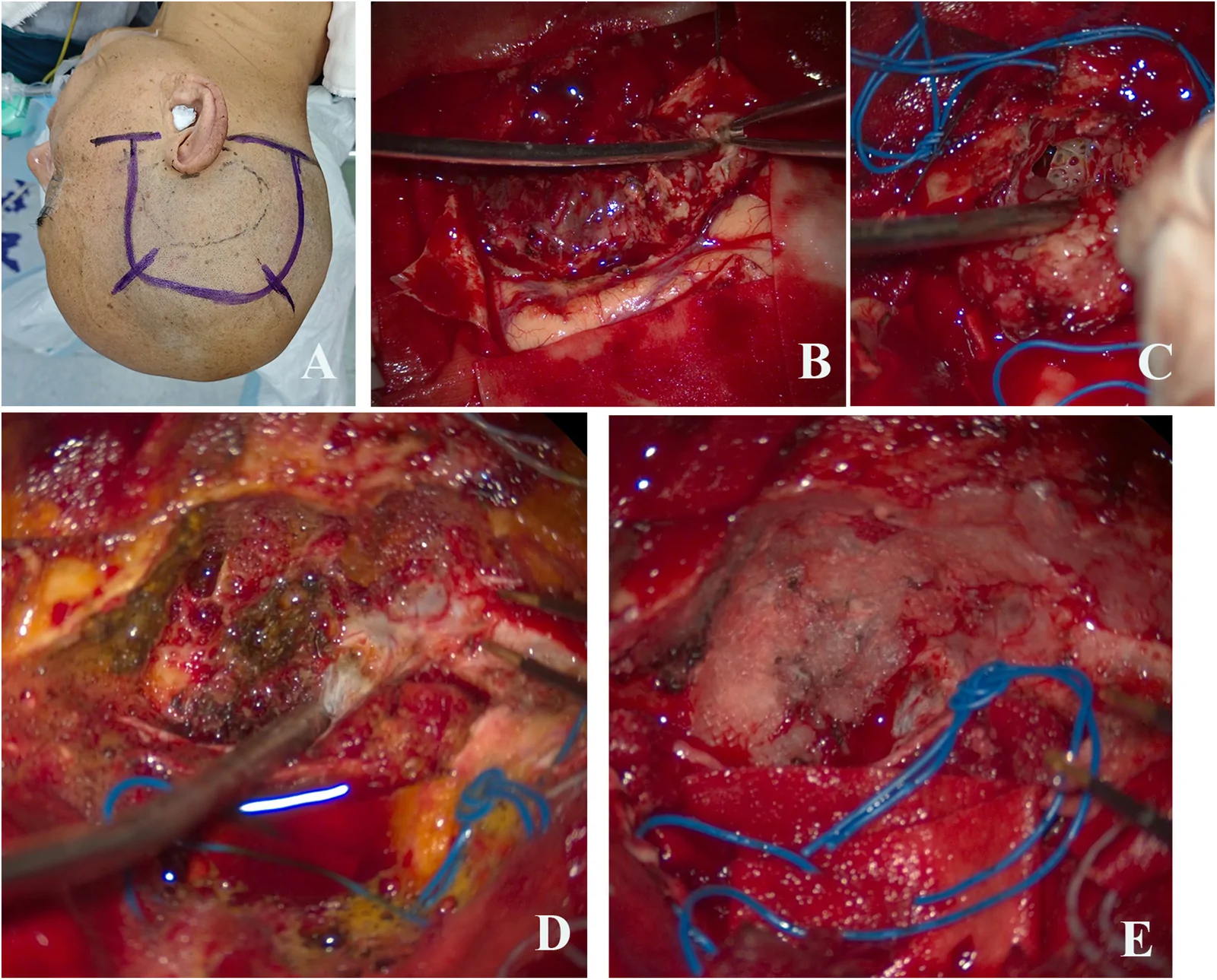
Surgical procedure. **(A)** Skin incision. **(B)** Resection of the temporal lobe tumor. **(C)** The tumor invaded the mastoid process, and the lesion was carefully dissected away from the mastoid. **(D)** Resection of the tumor involving the mastoid process. **(E)** The mastoid air cells were meticulously sealed with bone wax.

Given the mass effect, rapid symptom progression, and radiological suspicion of an aggressive malignant lesion, the patient underwent surgical resection. Intraoperatively, the tumor was located in the right temporal lobe and was found to extend to the dura with contiguous involvement of the adjacent calvarial lesion. Gross total resection of the intracranial tumor was achieved, together with removal of the involved skull lesion. Cranioplasty was subsequently performed. The lesion appeared highly vascular and invasive, but no gross purulent material or alternative pathology was encountered (Figures 2A–E).

**Figure 3:**
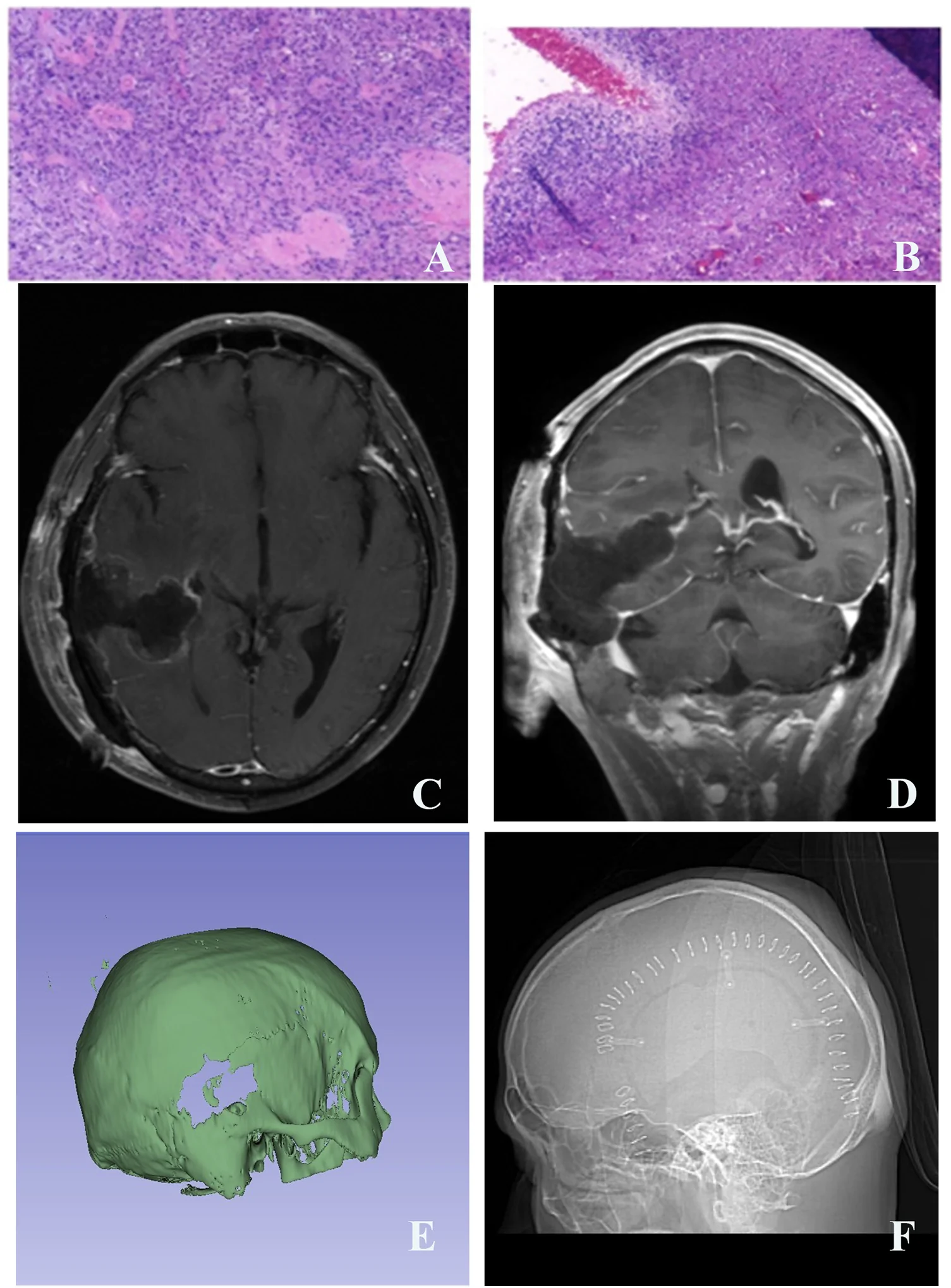
Histopathological findings AND postoperative follow-up MRI. **(A)** Low-magnification histopathological view. **(B)** High-magnification histopathological view. **(C,D)** Postoperative contrast-enhanced T1-weighted axial and coronal MRI scans confirmed gross total resection of the tumor. **(E)** preoperative CT demonstrating calvarial destruction, 3D volume-rendered reconstruction illustrating the extent of skull defect. **(F)** Replacement of bone flap after operation.

Histopathological examination demonstrated a highly cellular astrocytic neoplasm with marked nuclear atypia, brisk mitotic activity, microvascular proliferation, and necrosis, consistent with glioblastoma, CNS WHO grade 4 (Figures 3A,B). Immunohistochemistry supported glial differentiation, including positivity for glial lineage markers. The integrated molecular profile was consistent with IDH-wildtype glioblastoma and showed the following alterations: TERT promoter mutation, CDKN2A/CDKN2B homozygous deletion, PTEN loss, chromosome 10 loss, MGMT promoter unmethylation, and absence of 1p/19q codeletion. These findings fulfilled contemporary diagnostic criteria for IDH-wildtype glioblastoma and were compatible with a biologically aggressive tumor (14–17).

Postoperatively, the patient recovered well. Early postoperative contrast-enhanced MRI indicated gross total resection. Headache improved significantly after surgery, and no new neurological deficits developed. The patient remained free of wound infection and cerebrospinal fluid leakage, and he was able to ambulate independently at discharge (Figures 3C,D).

He subsequently received standard adjuvant chemoradiotherapy consisting of radiotherapy to the tumor bed (60 Gy in 30 fractions) and surrounding low-risk field (54 Gy in 30 fractions) with concurrent temozolomide, followed by adjuvant temozolomide, which is still ongoing at the time of manuscript preparation. At the 3-month postoperative follow-up, the patient was alive, clinically stable, and without radiographic evidence of tumor progression.

The patient and his family reported significant improvement in neurological symptoms following surgery, particularly in headache relief and gait stability. The patient expressed satisfaction with the surgical outcome and the subsequent recovery process, noting improved ability to perform daily activities independently. He is currently continuing adjuvant chemoradiotherapy and remains under regular outpatient follow-up. The patient and family have been informed regarding the aggressive nature of glioblastoma and the need for long-term surveillance.

## Discussion

Glioblastoma (GBM) is characterized by highly infiltrative growth within the central nervous system, but direct transdural extension with contiguous invasion of the calvarium or skull base remains exceptionally uncommon (1–6). Most reports of extra-cranial spread in GBM concern hematogenous metastasis or dissemination following surgery, biopsy, or radiotherapy, whereas spontaneous osseous invasion by a previously untreated primary intracranial GBM is rare (3–6). In the present case, the tumor exhibited transdural extension with destruction of the temporal calvarium and adjacent mastoid region at initial presentation, without any prior cranial intervention. This pattern not only expands the recognized anatomical spectrum of GBM invasion, but also raises important questions regarding the mechanisms, diagnostic evaluation, and clinical implications of this unusual phenotype.

Differentiating GBM from extra-axial lesions is critical. Cystic meningiomas may show heterogeneous enhancement and cystic degeneration but usually demonstrate dural origin, dural tail sign, and hyperostotic bone reaction rather than osteolysis (19). Intracranial solitary fibrous tumor/hemangiopericytoma (SFT/HPC) often presents as a highly vascular extra-axial mass with intense enhancement, flow voids, and aggressive but typically extra-axial growth pattern (20). In contrast, the present case showed a clearly intra-axial origin with secondary dural and bone invasion. Magnetic resonance spectroscopy further supported a glial origin, showing elevated choline and lipid/lactate peaks, more consistent with GBM than meningioma or SFT/HPC (21).

To place the current case in context, we reviewed previously reported cases of glioblastoma with spontaneous transdural extension and contiguous skull invasion (Table 1). Although the available literature is limited and largely based on isolated case reports, several recurring observations can be identified (7–12). First, many reported lesions were superficially located and maintained broad contact with the dura, particularly in the temporal or frontotemporal region (8–12). Such anatomical proximity may facilitate progressive disruption of the meningeal barrier and eventual extension into adjacent bone. Second, radiological interpretation is often challenging, because skull destruction or extracranial soft tissue extension more commonly suggests metastatic disease, aggressive meningioma, or primary skull malignancy rather than an intra-axial glioma (7–12). Third, most historical reports predate contemporary molecular classification and therefore provide limited insight into whether this invasive phenotype reflects a distinct biological subset of GBM or simply an uncommon anatomical manifestation of otherwise conventional IDH-wildtype glioblastoma.

**Table 1:** Representative reported cases of glioblastoma with spontaneous transdural and skull-invasive presentation.

Study	Age/sex	Tumor location	Dural/skull involvement	Molecular information	Treatment/outcome
Pompili et al., 1993 (8)	Adult/NR	Supratentorial glioma(s)	Transdural extension through dura	Not available	Historical surgical management; limited outcome detail
Rainov et al., 1996 (9)	Adult/male	Temporal glioblastoma	Skull-base destruction with direct extracranial extension	Not available	Surgery reported; locally aggressive skull-invasive GBM
Gheyi et al., 2004 (10)	Adult/NR	Cerebral GBM abutting calvarium	Calvarial destruction by GBM	Not available	Surgical management; radiologic-pathologic confirmation
Satyarthee and Mahapatra, 2015 (11)	Pediatric/male	Giant supratentorial GBM	Primary calvarial erosion with sutural diastasis	Not available	Gross resection reported; pediatric skull-invasive presentation
Thrull et al., 2023 (12)	Adult/female	Temporal/skull-base region	Transdural skull-base infiltration with extracranial extension	Limited molecular information	Surgery plus adjuvant treatment
Present case	63/male	Right temporal lobe	Temporal calvarial destruction + mastoid osteolysis	IDH-wildtype; TERT promoter mutation; CDKN2A/CDKN2B homozygous deletion; PTEN loss; MGMT unmethylated	GTR + RT/TMZ; alive without progression at 3 months

The mechanism by which GBM penetrates the dura and invades bone remains incompletely understood. Under normal circumstances, the dura mater, skull, and associated connective tissue structures represent substantial barriers to extra-axial spread. However, superficially located tumors that remain in prolonged contact with the dura may gradually infiltrate the leptomeninges and dural interface, particularly when driven by an intrinsically invasive tumor phenotype (8–12). In the present case, the lesion arose in the right temporal lobe and was closely apposed to the adjacent temporal dura and skull, which may have created favorable anatomical conditions for transdural extension.

An important issue is whether the molecular profile of this case truly distinguishes it from conventional GBM. The molecular findings in our patient—namely IDH-wildtype status, TERT promoter mutation, CDKN2A/CDKN2B homozygous deletion, PTEN loss, chromosome 10 loss, absence of 1p/19q codeletion, and MGMT promoter unmethylation—are largely consistent with the canonical profile of adult IDH-wildtype glioblastoma rather than indicating a clearly distinct skull-invasive subtype (14–18). Accordingly, this case does not establish a unique molecular category of GBM characterized by transdural and osseous spread.

From a clinical perspective, the present case is notable not only because of its rarity, but also because it provides a contemporary, molecularly characterized example of spontaneous skull-invasive GBM accompanied by postoperative treatment and follow-up data. Gross total resection of the intracranial lesion and involved skull was followed by radiotherapy to the tumor bed (60 Gy/30 fractions) and surrounding low-risk field (54 Gy/30 fractions) with concurrent temozolomide, followed by adjuvant temozolomide. Early postoperative MRI indicated gross total resection, and at 3-month follow-up the patient remained alive without radiographic progression, with improvement of headache and no new neurological deficits.

## Limitations

This report has several limitations. First, as a single case, it cannot determine whether skull-invasive GBM represents a reproducible molecular subgroup or merely an uncommon anatomical growth pattern of conventional IDH-wildtype glioblastoma. Second, the follow-up duration is currently short, limiting assessment of long-term local control, progression pattern, and survival. Third, no transcriptomic or proteomic analyses were available to directly evaluate invasion-associated pathways such as matrix remodeling, mesenchymal transition, or osteolytic signaling. Finally, the literature on spontaneous skull-invasive GBM is sparse and heterogeneous, and many published cases predate contemporary WHO molecular classification, which limits cross-study comparison.

## Conclusion

We report a rare case of primary right temporal IDH-wildtype glioblastoma presenting with spontaneous transdural invasion, temporal calvarial destruction, and contiguous mastoid osteolysis prior to any cranial intervention. This case broadens the recognized clinicoradiological spectrum of glioblastoma and highlights that skull destruction does not exclude an intra-axial glial malignancy. By providing integrated MRI/MRS, histopathology, molecular profiling, postoperative chemoradiotherapy details, and short-term follow-up, this report adds clinically relevant information to the limited literature on skull-invasive glioblastoma.

## Data Availability

The datasets presented in this study can be found in online repositories. The names of the repository/repositories and accession number(s) can be found in the article/Supplementary Material.
